# The complete mitochondrial genome of the Australian Common Rock Rat, *Zyzomys argurus*

**DOI:** 10.1080/23802359.2021.1920858

**Published:** 2021-07-27

**Authors:** Jaco D. Zandberg, Wayne G. Reeve, Serina McConnell, Peter B. S. Spencer

**Affiliations:** a Medical, Molecular and Forensic Sciences, Murdoch University, Murdoch, Australia; b Environmental and Conservation Sciences, Murdoch University, Murdoch, Australia

**Keywords:** Chordata, common Rock Rat, D-loop, mitogenome, Rodentia, Western Australia

## Abstract

The Common Rock Rat *Zyzomys argurus* is an abundant small- to medium-sized Murid rodent that is endemic to Australia. It is a nocturnal mammal with a mostly herbivorous diet. This species is native to the wet/dry tropics of Northern Australia and can be identified from other rock rats on the basis of its small size and its tail length (which is at least equivalent to its head-body length). Here, we describe the complete mitochondrial genome of *Z. argurus* and compare it to other Rodentia. The *Z. argurus* circular mitogenome was 16,261 bp and contained 13 protein-coding genes, two rRNA genes, 22 tRNAs and a control region (D-loop) of 859 bp. Phylogenetic analysis of selected, published sequenced mitogenomes reveal it is most closely related to the Lakeland Downs mouse *Leggadina lakedownensis* in the order Rodentia.

Australian rock rats are saxicoline Murid rodents that belong to the endemic genus *Zyzomys*. There are five species that have been identified in the *Zyzomys* genus, including the Common Rock Rat *Zyzomys argurus* (Thomas, 1889), which shares similar features with the other species but is notably smaller (Van Dyck and Strahan 2008). *Zyzomys argurus* is widely distributed in the Northern tropical regions of Australia (WA, NT, and QLD) with seasonal fluctuations in abundance. These rodents are nocturnal and typically found in rocky ranges and outcroppings. They feed mostly on plants, seeds, fungi, and occasionally on insects. The species is thought to store fat at the base of the tail for use during lean times (Begg 1981) which occur particularly in the wet season. If necessary, they can shed the tail skin (autotomic skin release) to escape predation, a situation also observed for two species of African spiny mice (Seifert et al. 2012). Morphologically, the Common Rock Rat can be distinguished from other rock rats by the length of its tail (tail = head–body length), its small size, lighter color and sparsely haired tail. After 5–6 months, the adults are sexually mature, and females are distinguished by having four nipples to feed their young (Fleming 2008). Aside from morphological features that can be used for classification, it is evident from many studies that mtDNA can make a significant contribution to phylogenetics, allowing for the establishment of the evolutionary history of a taxon and which can complement classification based on nuclear DNA markers (Gupta et al. 2015). To our knowledge, the mitochondrial genome has not been established for any species of *Zyzomys*. In this study, we have, therefore, determined the complete mitochondrial genome for *Z. argurus* to enable genetic comparisons to be made. The *Z. argurus* DNA was sourced from voucher material from Scott Strait in the Kimberley region of Western Australia (−14.6067 S; 125.2572 E; lab number 17-108). WA Museum voucher number M56078.

The DNA was sequenced using the Illumina MiSeq Platform (Illumina, San Diego, CA) which produced 5,558,506 paired-reads reads totaling 1.668 Gbp. The 300 bp paired-end reads were trimmed by BBDuck at Q13 and then merged using the Geneious Prime v2019.2.1 (Biomatters^®^, NZ) internal merging tool resulting in 1,207,872 curated reads. We then *de novo* assembled the complete mitogenome using 11,354 reads (0.94% of the curated reads) totaling 3,398,549 bp, to produce a circular mitogenome of size 16,261 bp with 209× coverage. Annotations for the protein-coding, tRNA, and rRNA genes for the finished mitogenome were retrieved from the published Lakelands Downs Mouse mitogenome using the “Annotate and Predict” feature of Geneious Prime v2019.2.1 (Biomatters^®^, NZ). The sequence with annotated features has been deposited in GenBank under the Accession no. MT741674. The *Z. argurus* complete mitogenome sequence length was established to be 16,261 bp with a typical vertebrate mitogenome organization (Nilsson et al. 2004; Westerman et al. 2016) containing 13 protein-coding genes, 2 rRNA genes, 22 tRNA genes, and a non-coding control region (D-loop). The overall base composition was 35.8% A, 30.6% T, 22% C, and 11.6% G, with a GC% content of 33.6% which is similar to other Murid mitochondrial genomes. Of the 13 protein-coding genes, 10 initiated with ATG, while 2 started with ATA (ND2 and ND3) and 1 with ATT (ND5). Twelve protein-coding genes ended with TAA; eight of which had TAA as the stop codon in the gene sequence (ATP6, ATP8, COX1, COX2, ND3, ND4L, ND5 and ND6) and the other four had the stop codon completed by the addition of 3′ A-residues to the mRNA (COX3, CYTB, ND2, and ND4). One gene, ND1, had a TAG stop codon.

Phylogenetic analysis was conducted using slowly evolving mitogenome sequences (excluding tRNA, intergenic regions, and control regions) of 13 finished mitogenomes (Figure 1).The phylogenetic analysis conducted used the general time reversal (GTR) model (+G + I) of best fit in the program MEGA-X (Nei and Kumar 2000). As can be seen in Figure 1, the phylogenetic analysis revealed that *Z. argurus* is a member of the Rodentia order, with its closest relative being *Leggadina lakedownensis*. Furthermore, it is shown to be more closely related to a mouse than a rat.

**Figure 1.**
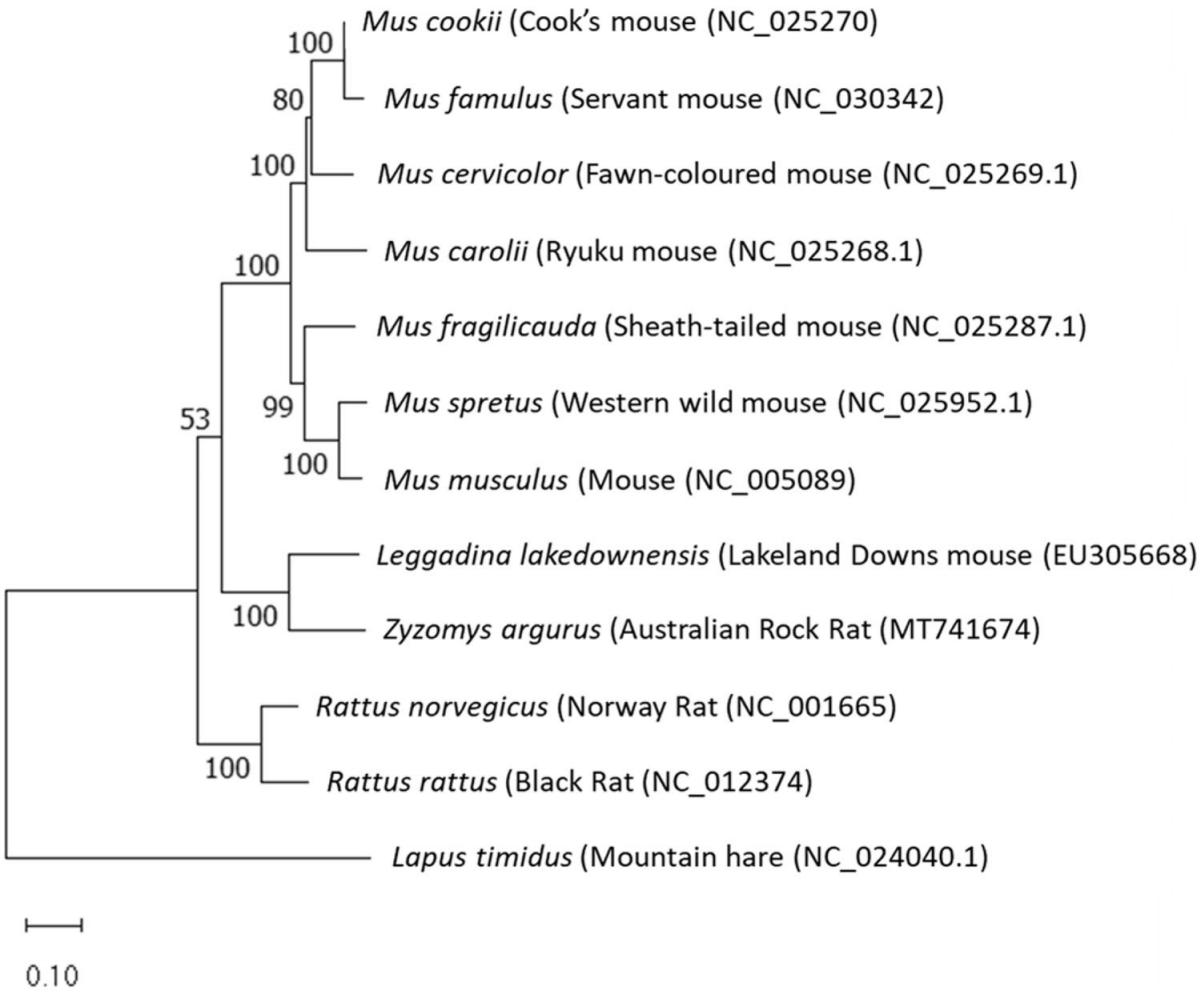
Phylogenetic placement of *Zyzomys argurus* based on a truncated comparison of the rRNA and coding DNA sequences to other entire vertebrate mitogenomes (references for the genomes can be found in NCBI accessions). The evolutionary history was inferred by using the maximum-likelihood method and General Time Reversible model (Nei and Kumar 2000). The tree with the highest log likelihood (−71,921.01) is shown. The percentage of trees in which the associated taxa clustered together is shown next to the branches. Initial tree(s) for the heuristic search were obtained automatically by applying Neighbor-Join and BioNJ algorithms to a matrix of pairwise distances estimated using the maximum composite likelihood (MCL) approach, and then selecting the topology with superior log likelihood value. A discrete Gamma distribution was used to model evolutionary rate differences among sites (5 categories (+G, parameter = 0.3903)). The rate variation model allowed for some sites to be evolutionarily invariable ([+I], 29.14% sites). The tree is drawn to scale, with branch lengths measured in the number of substitutions per site. This analysis involved 12 nucleotide sequences. There were a total of 13,603 positions in the final dataset. Evolutionary analyses were conducted in MEGA X (Kumar et al. 2018).

## Geolocation information

Geospatial coordinates from voucher material for the Common Rock Rat (*Zyzomys argurus*) originated from Scott Strait in the Kimberley region of Western Australia (−14.6067 S; 125.2572 E; lab number 17-108). WA Museum voucher number M56078.

## Data Availability

The data that support the findings of this study are available from either GenBank (see Figure 1) or from the corresponding author (P. B. S. S.) upon reasonable request. The complete mitochondrial sequence has been deposited in the NCBI online database at https://www.ncbi.nlm.nih.gov/nuccore/MT741674.
